# Correlative Assembly of Subsynaptic Nanoscale Organizations During Development

**DOI:** 10.3389/fnsyn.2022.748184

**Published:** 2022-05-24

**Authors:** Shi-Yan Sun, Xiao-Wei Li, Ran Cao, Yang Zhao, Nengyin Sheng, Ai-Hui Tang

**Affiliations:** ^1^Chinese Academy of Sciences (CAS) Key Laboratory of Brain Function and Disease, Ministry of Education Key Laboratory for Membrane-less Organelles and Cellular Dynamics, Division of Life Sciences and Medicine, University of Science and Technology of China, Hefei, China; ^2^Institute of Artificial Intelligence, Hefei Comprehensive National Science Center, Hefei, China; ^3^State Key Laboratory of Genetic Resources and Evolution in Kunming Institute of Zoology, Chinese Academy of Sciences, Kunming, China

**Keywords:** nanocluster, super-resolution, STORM, glutamate receptor, nanocolumn

## Abstract

Nanoscale organization of presynaptic proteins determines the sites of transmitter release, and its alignment with assemblies of postsynaptic receptors through nanocolumns is suggested to optimize the efficiency of synaptic transmission. However, it remains unknown how these nano-organizations are formed during development. In this study, we used super-resolution stochastic optical reconstruction microscopy (STORM) imaging technique to systematically analyze the evolvement of subsynaptic organization of three key synaptic proteins, namely, RIM1/2, GluA1, and PSD-95, during synapse maturation in cultured hippocampal neurons. We found that volumes of synaptic clusters and their subsynaptic heterogeneity increase as synapses get matured. Synapse sizes of presynaptic and postsynaptic compartments correlated well at all stages, while only more mature synapses demonstrated a significant correlation between presynaptic and postsynaptic nano-organizations. After a long incubation with an inhibitor of action potentials or AMPA receptors, both presynaptic and postsynaptic compartments showed increased synaptic cluster volume and subsynaptic heterogeneity; however, the trans-synaptic alignment was intact. Together, our results characterize the evolvement of subsynaptic protein architectures during development and demonstrate that the nanocolumn is organized more likely by an intrinsic mechanism and independent of synaptic activities.

## Introduction

Synapses are highly diverse and plastic in morphology and function (Walmsley et al., 1998). Even for the same type, the sizes of both presynaptic and postsynaptic compartments can vary by two orders of magnitude (Harris and Stevens, 1989). Subsynaptic organizations of synaptic proteins have conducted a new layer of structural and functional heterogeneity. Key proteins in the presynaptic and postsynaptic compartments, including RIM, Munc13, receptors, and several postsynaptic scaffolds, are organized in the form of nanoscale assemblies with a similar size of ~100 nm, namely, nanoclusters or nanodomains (Fukata et al., 2013; MacGillavry et al., 2013; Nair et al., 2013; Hruska et al., 2018; Kellermayer et al., 2018; Sakamoto et al., 2018). More importantly, these nanoclusters are coupled spatially within nanocolumns so that the presynaptic RIM nanoclusters could guide the action potential-dependent transmitter release to take place preferentially on postsynaptic receptor densities (Tang et al., 2016; Petzoldt et al., 2020). This arrangement and its reorganization could play a major role in controlling the efficacy and plasticity of synaptic transmissions (Sinnen et al., 2017; Chen et al., 2018; Groc and Choquet, 2020), as has been predicted by numerical studies (Raghavachari and Lisman, 2004; MacGillavry et al., 2013). In fact, during synaptic plasticity, these subsynaptic nano-organizations undergo vigorous remodeling (Tang et al., 2016; Hruska et al., 2018). Mechanisms have been proposed for their formation and maintenance: synaptic proteins can undergo self-assembly to form condensates through phase separation (Zeng et al., 2018, 2019; Wu et al., 2019), and the alignment between presynaptic and postsynaptic nanoclusters is mediated by synaptic adhesion molecules including neuroligin1 (Haas et al., 2018) and LRRTM2 (Ramsey et al., 2021). However, it remains to be demonstrated how these nanostructures are formed in real synapses.

Early electron microscopy studies and fluorescence imaging have demonstrated a strong correlation between structural features including bouton volume, active zone (AZ) area, postsynaptic density (PSD) area, spine volume, and number of receptors and key scaffolds (Harris and Stevens, 1989; Schikorski and Stevens, 1997; Nusser et al., 1998; Takumi et al., 1999; Regalado et al., 2006; Kay et al., 2011; Holderith et al., 2012; Fisher-Lavie and Ziv, 2013; Rollenhagen et al., 2018). These correlations between structural features correspond well to the functionality of synapses. Two-photon Ca^2+^ imaging and uncaging studies revealed that presynaptic release probability scales well with the AZ size, and larger spines show larger postsynaptic responses (Matsuzaki et al., 2001; Holderith et al., 2012). Recently, with combined slice electrophysiology and correlated light microscopy and high-resolution EM, Holler et al. demonstrated a strong linear relationship between synaptic strength and PSD area (Holler et al., 2021). Consistently, further functional studies also demonstrated a correlation between presynaptic release probability and postsynaptic AMPAR abundance or EPSCs (Thiagarajan et al., 2005; Tokuoka and Goda, 2008; Kay et al., 2011). The existence of nanocolumns strongly suggests a trans-synaptic correlation between nano-organizations. However, only half of the nanoclusters are actually coupled within nanocolumns (Tang et al., 2016), whether the general heterogeneity of protein organizations in the two compartments is matched and how it is coordinated during development have not been studied.

In this study, by employing the stochastic optical reconstruction microscopy (STORM) and sophisticated analytical methods, we set out to study the nanoscale protein architecture of individual synapses in dissociated hippocampal neurons to establish how key synaptic proteins, including RIM1/2, AMPAR, and PSD-95, are subsynaptically organized during early development. We found that the subsynaptic heterogeneity of synaptic proteins was not inherent but gradually organized during development. The protein nano-organizations in presynaptic and postsynaptic compartments were largely correlated and developed coordinately with no evidence of one side leading the other. Moreover, on synaptic activity blockade, both presynaptic and postsynaptic compartments showed an increased cluster size and subsynaptic heterogeneity, and the trans-synaptic alignment remained intact. Together, our results characterize the evolvement of subsynaptic protein architectures during development and suggest an intrinsic self-organization mechanism for the formation of nanocolumn organizations.

## Materials and Methods

### Neuron Culture

Dissociated hippocampal neuron cultures were prepared from E18 rat embryos and plated with a density of 50k per well on poly-lysine coated glass coverslips in a 12-well plate. All procedures conformed to the guidelines established by the Institutional Animal Care and Use Committees at the University of Science and Technology of China (USTC) and the Chinese Academy of Sciences (CAS). The activity blockade was performed by the addition of tetrodotoxin (TTX, 0.5 μM) or 2,3-dihydroxy-6-nitro-7-sulfamoylbenzo(f)-quinoxaline (NBQX, 10 μM) at DIV10. To make sure the blockade was effective through the long period, we added another dose at DIV14. Whenever the cultures were fed, TTX or NBQX was included in the fresh medium.

### Immunostaining

Cells were fixed with 4% paraformaldehyde (PFA) and 4% sucrose in phosphate-buffered saline (PBS) (pH 7.4) for 10 min at room temperature (RT), followed by washing with 50 mM glycine in PBS. Cells were then permeabilized and blocked using 3% bovine serum albumin (BSA) or 5% donkey serum in PBS/Gly with 0.3% Triton X-100, followed by incubation with primary antibodies (3 h RT) and then secondary antibodies (1 h RT).

The following primary antibodies were used in this study: rabbit anti-RIM1/2 (1:500, Synaptic Systems 140213), mouse anti-PSD-95 (1:200, NeuroMab 75-028), and rabbit anti-GluA1 (1:500, Merck Millipore AB1504). For co-staining of GluA1 and RIM1/2, as both antibodies were from rabbits, staining was performed separately and the first primary antibody (GluA1) was blocked and converted with a goat anti-rabbit Fab fragment (JacksonImmuno 111-007-003) for 2 h at RT, and then recognized by secondary antibodies. The secondary antibodies were Alexa-647 conjugated donkey anti-Goat (JacksonImmuno 705-605-147), Alexa-647 conjugated donkey anti-rabbit (JacksonImmuno 711-605-152), and unconjugated donkey anti-mouse antibody (JacksonImmuno 715-005-151) or donkey anti-rabbit antibody (JacksonImmuno 711-005-152) labeled with Cy^TM^3B (Mono NHS Ester, GE Healthcare 16889934).

### STORM Imaging

Imaging was carried out on a Nikon ECLIPSE Ti2 inverted microscope equipped with a perfect focusing system and a 100 × /1.49 TIRF oil-immersion objective controlled using NIS-Elements AR 4.30.02 software. The typical incident power was ~40 mW (measured through the objective). Samples were imaged in a freshly made STORM imaging buffer containing: 50 mM Tris, 10 mM NaCl, 20% glucose, 56 μg/ml glucose oxidase (Sigma), 18 μg/ml catalase (Sigma), and 100 mM cysteamine (Sigma). To reduce background fluorescence while maximizing the depth of view, we adjusted the incident angle of the excitation beam to near but less than the critical angle and to achieve oblique illumination of the sample. Emission was collected using a CMOS camera (ORCA-Flash4.0, Hamamatsu) at a frame rate of 50 Hz and stored as images with a pixel size of 160 nm (with an 0.4× lens in the emission path). Z positions were determined by the ellipticity of the single peaks generated by a cylindrical lens (focal length 100 mm) with a precision of 40–50 nm. Total 50k images were collected for each channel. TetraSpeck beads (100 nm; Invitrogen) deposited on a coverslip were localized for generating the calibration curves. In our system, the average deviation of the bead localizations after t correction was 10–15 nm in *x/y* and 40–50 nm in *z*. Localization detection, calibration, and drift correction were performed using the NIS-Elements AR analysis 4.40.00 software. Localization coordinates were then rendered into images (pixel size of 5 nm) using a two-dimensional Gaussian kernel (σ = 20 nm) with custom routines in MATLAB (Mathworks).

### Electrophysiology

Whole-cell patch clamp was carried out with patch-clamp amplifiers (MultiClamp 700B, Axon Instruments) at RT. The data were acquired and analyzed using custom Igor Pro (WaveMetrics) programs. Intracellular pipette solution (pH 7.3) contained 136.5 mM potassium gluconate, 17.5 mM KCl, 9 mM NaCl, 1 mM MgCl_2_, 10 mM HEPES, 0.2 mM EGTA, 2 mM ATP-Mg, and 0.3 mM GTP-Na. External bath solution (pH 7.3) contained 150 mM NaCl, 3 mM KCl, 3 mM CaCl_2_, 2 mM MgCl_2_, 10 mM HEPES, 5 mM glucose, 1 μM TTX, and 20 μM bicuculline.

### Data Analysis

Detailed analysis on synaptic clusters was formed using custom routines in MATLAB as described previously (Tang et al., 2016; Chen et al., 2020). A synaptic cluster was identified in a 2D scatter plot of all localizations. By rotating a 3D scatterplot of localizations of a selected potential synaptic cluster pair, we evaluated the data quality and selected only those with clear edges (e.g., no nearby third cluster which may indicate more than two synaptic clusters in close proximity) for further analysis. To define a synaptic cluster on the random background, the nearest neighbor distances (NNDs) between localizations were calculated, and the mean + 2 × SD of NND was used as a cutoff to divide the localizations into subclusters. All localizations outside the primary subclusters were considered to be a background and were not used in further analysis. The border of the synaptic cluster was defined using the alpha-shape of the set of 3D localizations with α = 150 nm.

Nanoclusters were detected based on local density which was defined as the number of molecules within a radius of 2.5 times the standard median nearest neighbor distance (MdNND) for the calculation of the density of the synaptic cluster (Chen et al., 2020). The threshold of local density for nanocluster detection was defined as mean (LD0) + 4 × SD (LD0), where LD0 is the local density of a randomized cluster with the same overall density as the synaptic cluster. All localizations with a local density over this threshold were considered within nanoclusters. These localizations were then divided into subclusters with a “top-down” divisive strategy with a minimal peak-to-peak distance of 80 nm. Finally, to be counted as nanoclusters, those subclusters had to meet a set of criteria, including the number of localizations ≥8, which was derived empirically based on tests on our measured and simulated synapses to reduce the false positives arising from repeated localizations of the same molecule.

Data analysis was performed using the Origin software. Data are reported as average ± SEM values, and statistical significance was evaluated using one-way ANOVA, Kolmogorov–Smirnov test for cumulative curves, and *z*-test for proportions. Asterisks above brackets in data bar graphs indicate the level of statistical significance (^*^p < 0.05; ^**^p < 0.01; and ^***^p < 0.001). Detailed results of statistical analysis are listed in Supplementary Table 1.

## Results

### Correlative Expansion of Presynaptic and Postsynaptic Protein Clusters During Development

Previous ultrastructural and fluorescence imaging studies have shown a strong correlation between the sizes of membrane compartments and the amount of synaptic proteins on presynaptic and postsynaptic sides. In this study, we reexamined this with STORM super-resolution microscopy on presynaptic RIM1/2, the key AZ protein for action potential-dependent vesicle fusion, and postsynaptic AMPAR subunit GluA1 and scaffold PSD-95. We performed the staining and imaging as pairs of RIM1/2-GluA1 and RIM1/2-PSD-95, at four developmental stages in cultured hippocampal neurons: 6–8 days *in vitro* (marked as DIV7 in the following text) when synaptic contacts are newly formed, DIV10 (±1), DIV14 (±1), and DIV18 (±1) when synapses are thought mature.

**Figure 1:**
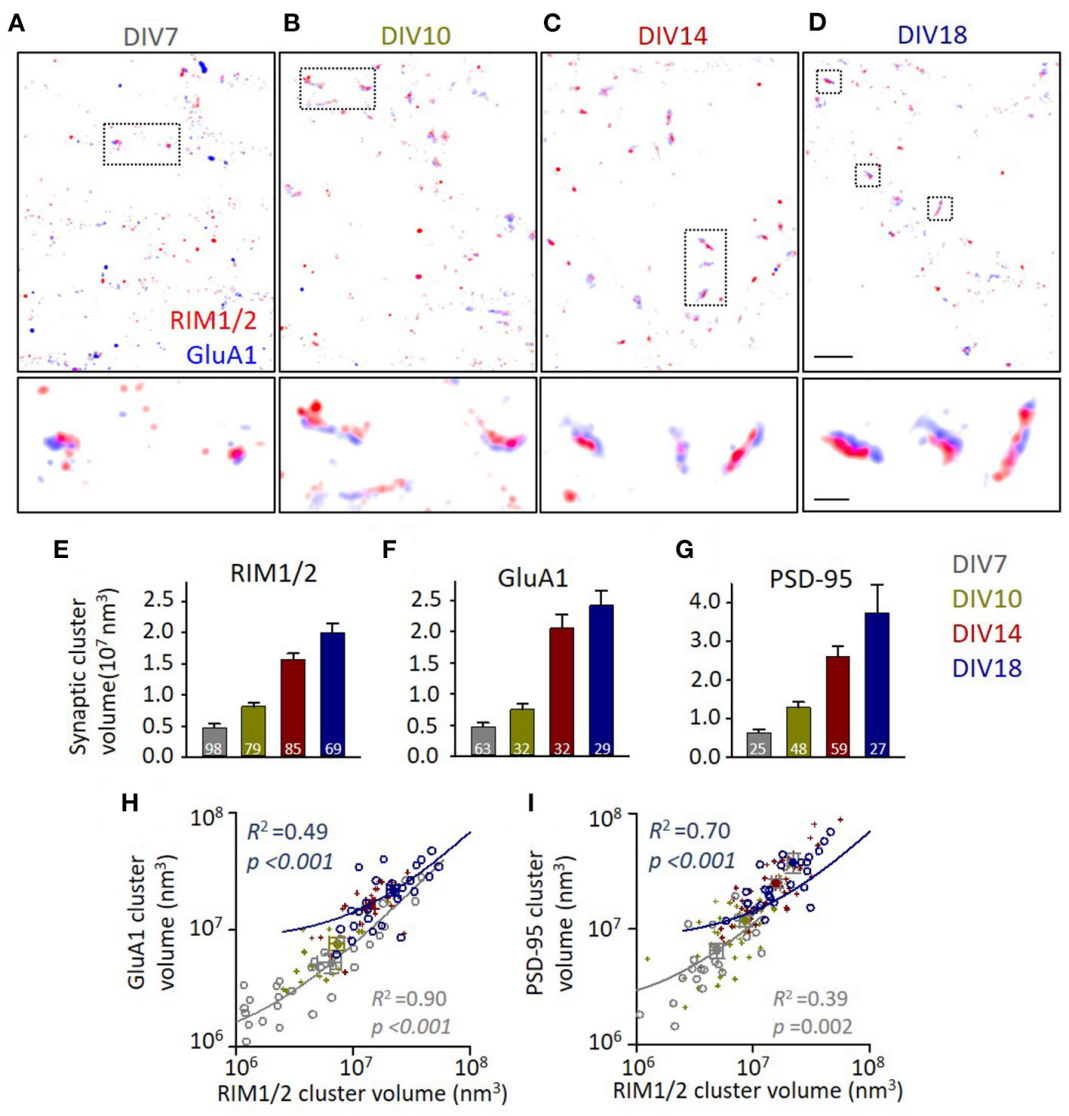
Synapse volumes increase correlatively during synaptic maturation. **(A–D)** Representative distribution of RIM1/2 and GluA1 under stochastic optical reconstruction microscopy (STORM). Scale 2 μm in top panels and 500 nm in lower panels. **(E–G)** Volumes of identified synaptic RIM1/2, GluA1, and PSD-95 clusters across different developmental stages. Numbers in bars denote the synapse numbers. **(H,I)** Correlations between the volumes of GluA1 and RIM1/2 clusters **(H)** and the volumes of PSD-95 and RIM1/2 **(I)** within the same synapses. Linear regressions were conducted on synapses of DIV7 (gray circles and line) and DIV18 (dark blue circles and line). Data from synapses of DIV10 and 14 were plotted with dark yellow and dark red crosses. Also refer to Supplementary Figures 1, 2 and Supplementary Table 1 for more details on correlations and statistics. All experiments were repeated ≥3 times.

At DIV7, synaptic contacts could be identified as sites of colocalization between RIM1/2 and GluA1 (or PSD-95), while there were many protein clusters without its counter partner in the vicinity (Figure 1A). Synaptic clusters were filtered, and the borders were defined with an alpha-shape as previously described (Tang et al., 2016; Chen et al., 2020). When the cultures grew older, the volume of synaptic clusters expanded gradually (Figures 1B–G), with a 4–5-fold increase in cluster sizes of all three proteins from DIV7 to DIV18 (*p* < 0.001 for all groups, one-way ANOVA; for details, refer to Supplementary Table 1), similar to previous observations (Chanda et al., 2017). More importantly, we found that this expansion in synaptic size was proportional between the presynaptic and postsynaptic compartments (Supplementary Figure 1). Actually, even at the same developmental state, the correlations between the volumes were strong for each protein (Figures 1H,I; Supplementary Figure 1). Moreover, a similar strong correlation was also found in the localization numbers of synaptic proteins which represent the amount of synaptic proteins (Supplementary Figure 2). These results suggest a synchronous growth of AZ and PSD during synapse maturation, and no obvious evidence indicating the remodeling in one compartment preceded that in the other.

### Evolvement of Subsynaptic Protein Nano-Organizations During Development

**Figure 2:**
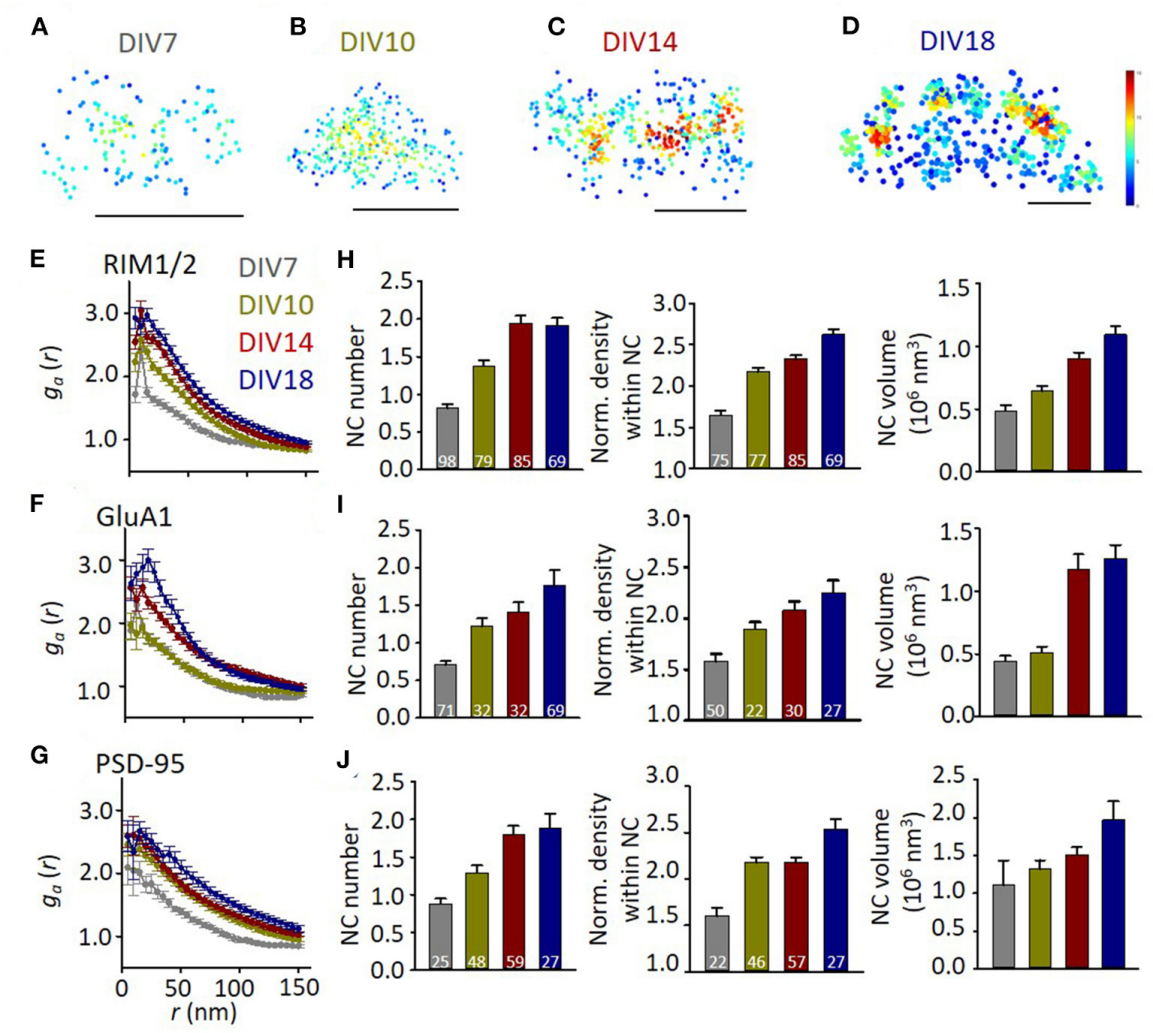
The heterogeneity of synaptic protein distribution increases with development. **(A–D)** Representative density maps of synaptic GluA1 at different developmental stages. Scale bars, 200 nm. **(E–G)** Normalized autocorrelation functions of RIM1/2 **(E)**, GluA1 **(F)**, and PSD-95 **(G)**. *g*_*a*_ above 1 suggests a significant non-uniform distribution. **(H–J)** Developmental changes of nanocluster number (left), normalized density within nanocluster (middle), and nanocluster volume of RIM1/2 **(H)**, GluA1 **(I)**, and PSD-95 **(J)**. Numbers in bars denote the synapse numbers. Also refer to Supplementary Table 1 for more statistical details. All experiments were repeated ≥3 times.

We then set out to quantify the subsynaptic distribution of these proteins at different developmental stages. To visualize the protein pattern within the synapse, local density for each localization was calculated and color-coded in the distribution map (Figures 2A–D). Comparing with that in mature synapses, GluA1 at DIV7-10 distributed more homogeneously within the synapse boundaries. To quantify the heterogeneity within the synaptic cluster, we employed a normalized autocorrelation function (*g*_*a*_) (Veatch et al., 2012; Tang et al., 2016). The autocorrelation showed a significant heterogeneity over a range of 0–100 nm for GluA1, RIM1/2, and PSD-95 at DIV14-18, while the amplitudes were significantly smaller and the range over which the heterogeneity was above the chance level was narrower for more immature synapses (Figures 2E–G). These differences were further confirmed by the properties of high-density nanoclusters identified with an automated algorithm based on local density (Chen et al., 2020). Immature synapses had a smaller number of nanoclusters, lower localization density within nanoclusters, and smaller nanocluster volume (Figures 2H–J). The gradually increased heterogeneity in these proteins during the maturation of synapses suggests that the well-organized subsynaptic distribution of both presynaptic transmitter release and postsynaptic receptors is a hallmark for mature synapses and may be essential for their functions.

**Figure 3:**
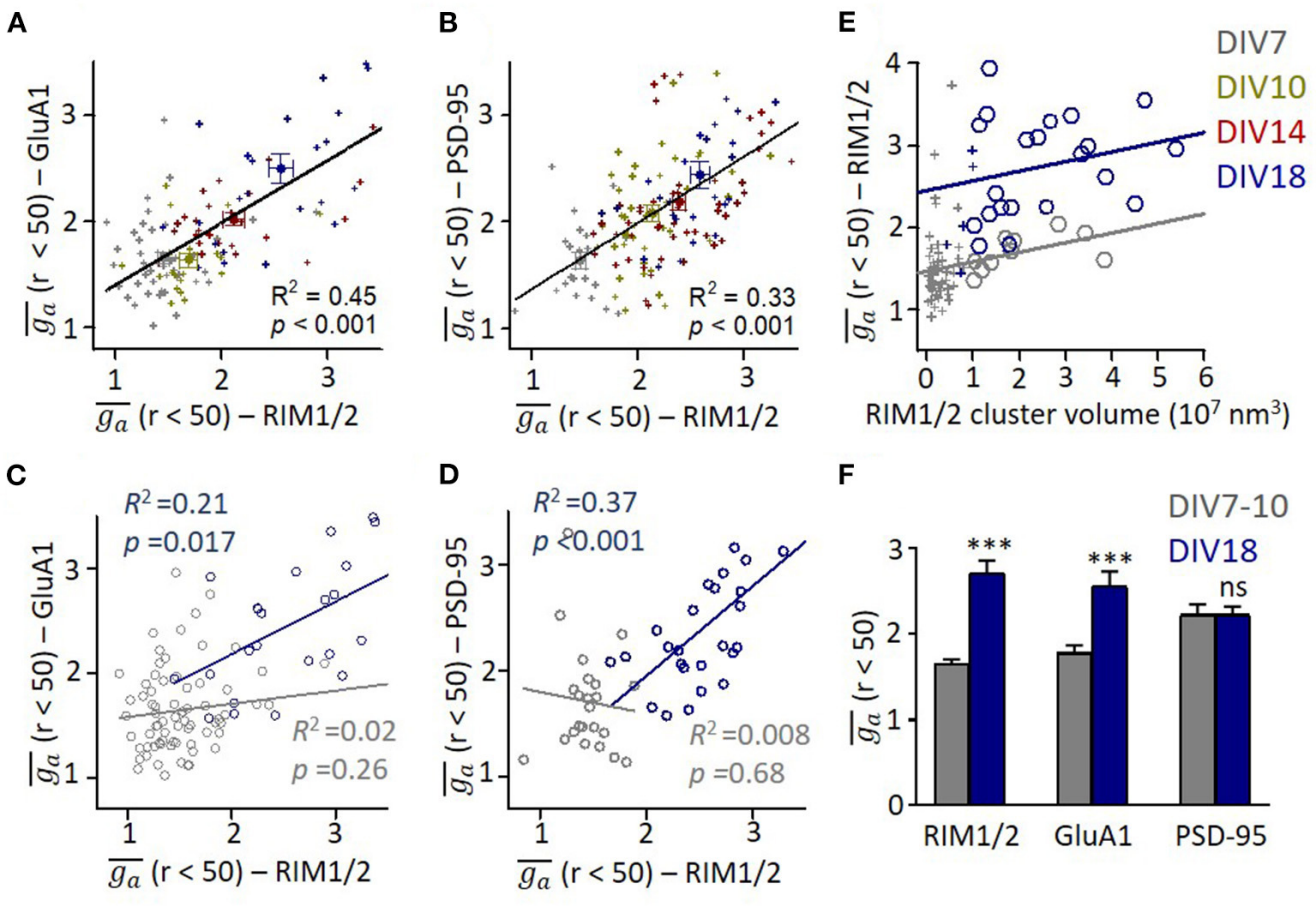
Correlation between presynaptic and postsynaptic protein heterogeneity in mature synapses. **(A,B)** Scatter plots for heterogeneity of GluA1 **(A)** and PSD-95 **(B)** against that of RIM1/2. All data points across all developmental stages could be fitted with linear functions as shown with lines. **(C,D)** Linear regressions of the relationships between heterogeneity of GluA1/PSD-95 and RIM1/2 at DIV7 (gray) and DIV18 (dark blue). **(E)** Relationship between heterogeneity and cluster volume of RIM1/2 at DIV7 (gray) and DIV18 (dark blue). Data points with cluster volume >1 × 10^7^ nm^3^ were fitted with linear functions. It is noted that *g*_*a*_ of immature synapses was significantly lower than that of matured synapses. **(F)** Averaged *g*_*a*_ of synapses with cluster volume of 1–4 × 10^7^ nm^3^ for immature (DIV7-10) and mature synapses (DIV18). Also refer to Supplementary Figure 3 for more details. ***p < 0.001, t-test. All experiments were repeated ≥3 times.

To compare the organizations on presynaptic and postsynaptic compartments, we averaged the *g*_*a*_ for radius from 0 to 50 nm ($\bar{g_{a}}$) and used it as a simplified index of subsynaptic heterogeneity. We found a significant correlation between $\bar{g_{a}}$ of presynaptic RIM1/2 and postsynaptic GluA1 or PSD-95 (*p* < 0.001 for both pairs, Figures 3A,B). This result suggests that the subsynaptic organizations of presynaptic and postsynaptic components are evolved synchronously during development, similar to the size of both compartments. When we examined the $\bar{g_{a}}\;$correlations in each developmental stage, we found that the correlation was only significant in mature synapses (Figures 3C,D). Since more mature synapses had a larger volume, we wonder whether stronger heterogeneity is an intrinsic property of larger synapses. We, therefore, plotted $\bar{g_{a}}$ of RIM1/2 against the cluster volume for each synapse and found that there was in fact a positive correlation (Figure 3E) for both mature and immature synapses. However, synapses of DIV18 showed a general larger $\bar{g_{a}}$ than those of DIV7. To eliminate the effect of cluster volume on $\bar{g_{a}}$, we picked up only those large synapses with a cluster volume of 1–4 × 10^7^ nm^3^ and compared the subsynaptic heterogeneity. The mature synapses showed a significantly larger $\bar{g_{a}}$ than the immature for both RIM1/2 and GluA1, but the heterogeneity of PSD-95 was similar (Figure 3F). These results suggest the subsynaptic heterogeneity of RIM1/2 or GluA1 is not an intrinsic property of the protein clusters, and there may be other active processes underlying the formation and evolvement of subsynaptic organizations during synaptic maturation.

### Development of Trans-Synaptic Release-Receptor Nano-Alignments

We previously showed that the presynaptic RIM1/2 nanoscale organization represents the preferential sites for transmitter release, and its alignment with postsynaptic AMPAR densities could efficiently modulate the synaptic strength (Tang et al., 2016). Therefore, it would be crucial to examine the nanoscale alignment between nanoclusters across the cleft during development.

**Figure 4:**
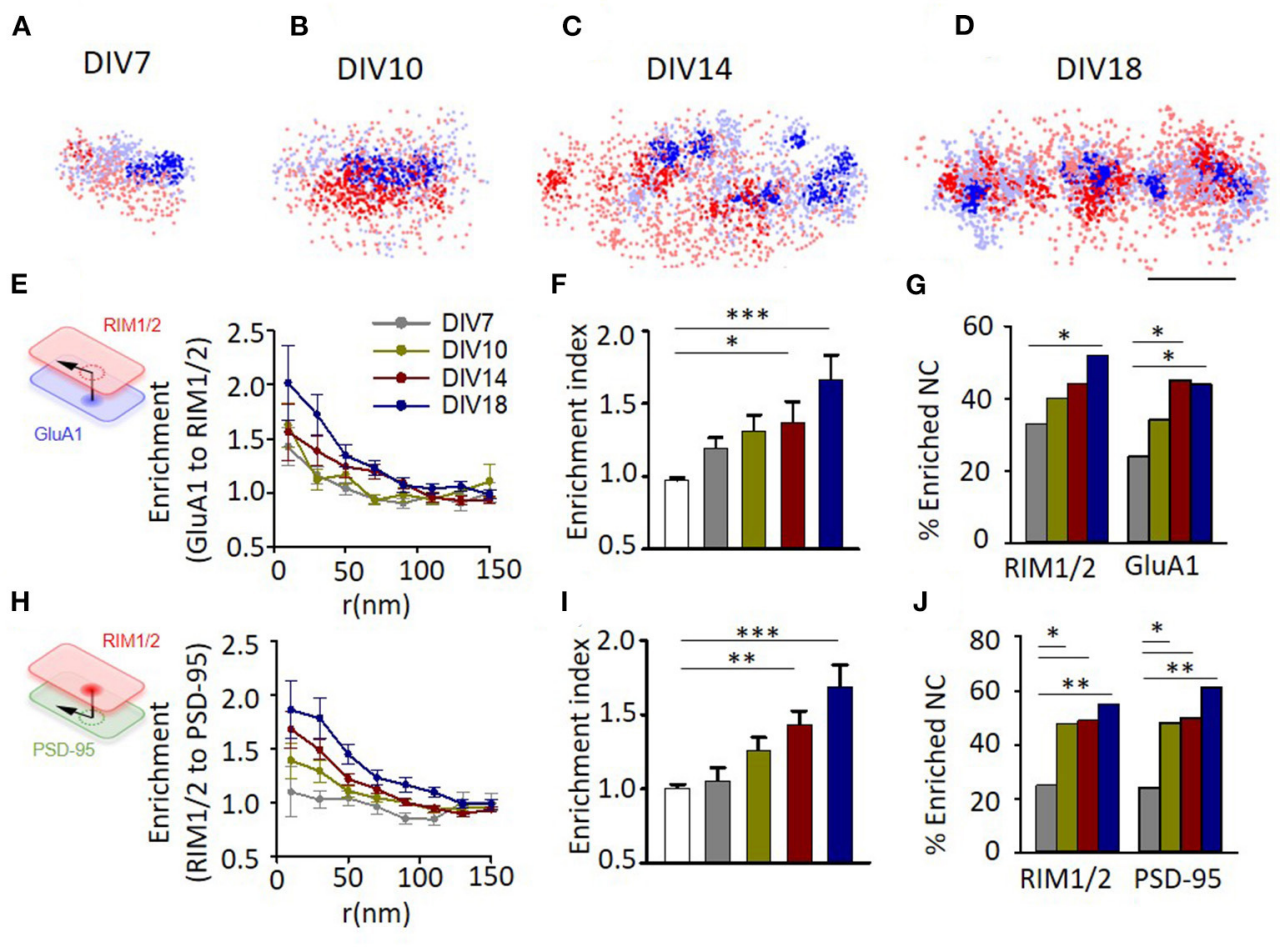
Evolvement of trans-synaptic nano-alignment during synaptic maturation. **(A–D)** Representative examples of synapses with RIM1/2 (red) and GluA1 (blue) co-labeled and imaged with STORM. Thick color denotes detected nanoclusters. Scale bar, 200 nm. **(E)** Normalized local density of GluA1 along with distances from RIM1/2 nanoclusters for synapses at different developmental stages. **(F)**, Averaged enrichment of GluA1 within 50 nm from peaks of RIM1/2 nanoclusters (*n* = 85, 60, 30, 54, and 54 nanoclusters). The open bar represents the enrichment indices of synapses with the position of nanoclusters randomized within synaptic clusters. **(G)** Fraction of nanoclusters that were enriched with protein across the cleft. **(H–J)** Enrichment between RIM1/2 and PSD-95 for synapses at different developmental stages. *p < 0.05, **p < 0.01, ***p < 0.001, one-way ANOVA with Tukey's multiple comparisons test in **(F,I)**, *z*-test in **(G,J)**. Also refer to Supplementary Table 1 for more statistical details. All experiments were repeated ≥3 times.

Representative examples showed that synapses at different developmental stages all have a certain degree of alignment between the nanoclusters of RIM1/2 and GluA1 (Figures 4A–D). To further quantify this, we performed the enrichment analysis by calculating the averaged local density of GluA1 along with different distances from the projected center of defined RIM1/2 nanoclusters (Figure 4E). All groups showed a trend of elevated GluA1 density at distances close to the RIM1/2 nanocluster centers, but more mature synapses exhibited a more significant enrichment. To simplify the comparison, we averaged the normalized GluA1 density within the distance of 50 nm to define an enrichment index (*EI*) and compared the measured indices with that of simulated synapses with the positioning of GluA1 nanoclusters randomized within the GluA1 cluster. Synapses in cultures of DIV14 and 18 showed an *EI* significantly above the randomized simulations (Figure 4F; *p* < 0.05, one-way ANOVA using the Tukey's multiple comparison test, for details, refer to Supplementary Table 1), while the *EI*s of immature synapses at DIV7 and 10 were not significantly different from the chance level.

By performing multiple simulations on the same synapse, we could get a threshold of *EI* (usually around 1.25–1.3) above which the tested RIM1/2 nanocluster was defined as a nanocluster with significant GluA1 enrichment, or simply “enriched nanocluster,” with a 95% CI (Tang et al., 2016). There were 33.3% of RIM1/2 nanoclusters above this threshold for synapses at DIV7, and the percentage increased gradually with development to 51.9% at DIV18 (Figure 4G). Similar results were found for the alignment between RIM1/2 and PSD-95 (Figures 4H,I), but one minor difference is that there were slightly more enriched nanoclusters at DIV10 compared with the GluA1-RIM1/2 pair (Figure 4J; 47.7% RIM1/2 and 48.1% PSD-95, vs. 10.0% RIM1/2, and 33.3% GluA1), suggesting the alignment between RIM1/2 and PSD-95 may form prior to that between RIM1/2 and GluA1.

### Activity-Dependency of Synaptic Nano-Architectures

**Figure 5:**
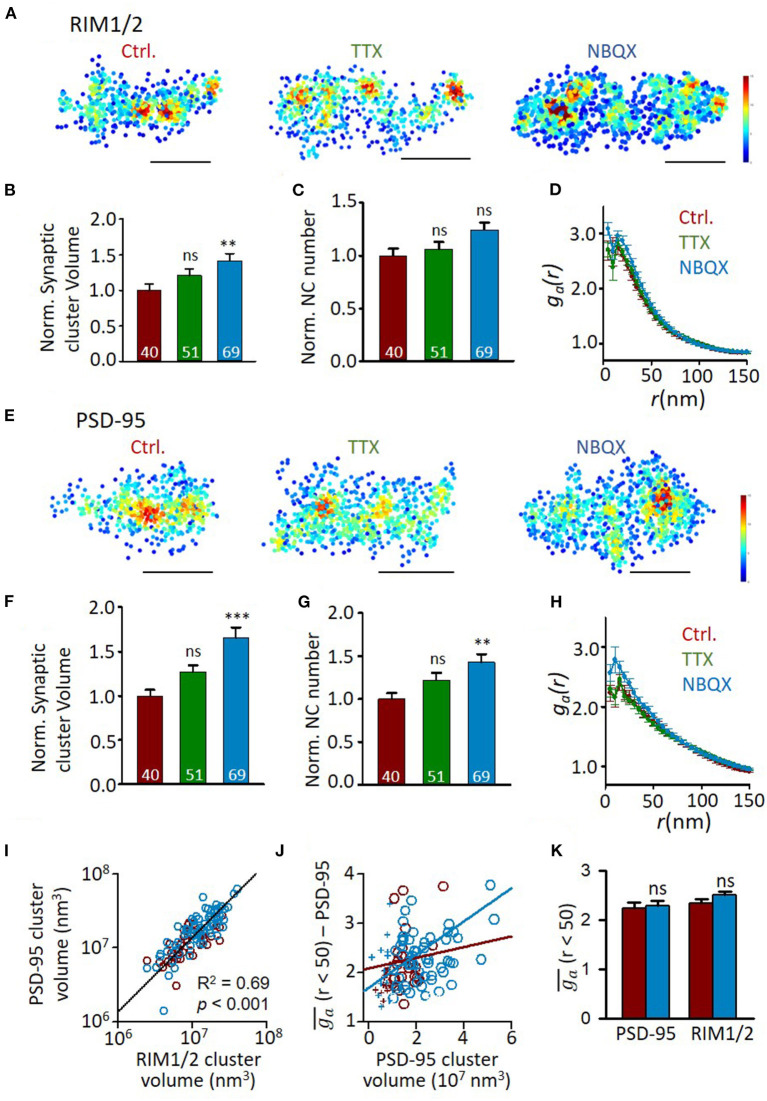
Effects of activity blockade on development of synaptic nano-organizations. **(A)** Representative density maps of RIM1/2 by STORM in cultures with different activity levels. Scale bar, 200 nm. **(B–D)** Comparison of synaptic cluster volume **(B)**, nanocluster number **(C)**, and auto-correlation profile **(D)** between RIM1/2 distributions from normal DIV18 neurons and cultures with activity blocked with TTX or NBQX. Numbers in bars denote the synapse numbers. **(E)** Example density maps of PSD-95 in cultures with different activity levels. Scale bar, 200 nm. **(F–H)** Properties of PSD-95 clusters and nanoclusters in synapses from normal DIV18 neurons and cultures with activity blocked with TTX and NBQX. **(I)** Correlation between the volumes of RIM1/2 and PSD-95 clusters within the same synapses. Linear regressions were conducted on synapses from all three groups. **(J)** Relationship between heterogeneity and cluster volume of PSD-95 in control (dark red) and NBQX group (blue). Data points with cluster volume >1 × 10^7^ nm^3^ were fitted with linear functions. **(K)** Averaged *g*_*a*_ of synapses with cluster volume of 1–3 × 10^7^ nm^3^ for control (dark red) and NBQX treated cultures (blue). **p < 0.01, ***p < 0.001, one-way ANOVA with Tukey's multiple comparisons. Also refer to Supplementary Table 1 for more statistical details. All experiments were repeated ≥3 times.

Previous studies found that the functional correlation between presynaptic and postsynaptic compartments requires neuronal activity to develop (Kay et al., 2011). We then sought to determine whether the correlation between nano-organizations was also dependent on synaptic activity. The hippocampal cultures were incubated in TTX (0.5 μM) or NBQX (10 μM) to block action potentials or AMPA receptors, respectively, at DIV10-18 during which the transsynaptic alignment and organizations show most dramatic changes (Figures 2, 4). This blockade lasted much longer than the treatment commonly used for homeostatic plasticity studies, and therefore, more processes may have taken place. In fact, though the frequency of miniature EPSCs showed a similar trend of increase after TTX incubation, the effects of both treatments were much smaller and insignificant than that of the previous 1–2 days' treatment (Han and Stevens, 2009) (Supplementary Figure 4). We found that AMPAR blockade significantly increased the synaptic cluster volume of both RIM1/2 and PSD-95 at DIV18 (Figures 5A,B,E,F) as well as the nanocluster number of PSD-95 (Figures 5C,G). Synapses in cultures treated with TTX showed a similar trend but the differences did not reach a significant level. These changes and trends were largely consistent with previous observations under similar treatment (MacGillavry et al., 2013; Glebov et al., 2017; Venkatesan et al., 2020).

However, the normalized density within nanoclusters showed no change with the treatment (Supplementary Table 1). Consistently, the autocorrelation profiles of either protein were largely overlapped (Figures 5D,H), except that PSD-95 showed slightly larger values of heterogeneity. To test whether this results from the larger cluster volume (Figures 3E, 5I), we similarly plotted $\bar{g_{a}}$ of PSD-95 against the cluster volume for each synapse and found that $\bar{g_{a}}$ of PSD-95 was, in fact, positively correlated with the cluster volume (Figure 5J). To rule out the effect of cluster volume, we selected only the synaptic clusters with a volume of (1–3) × 10^7^ nm^3^ and found that $\bar{g_{a}}$ of either PSD-95 or RIM1/2 showed no significant difference between control and AMPAR blockade (Figure 5K), suggesting that the general heterogeneity of synaptic proteins was largely unchanged by activity blockade.

**Figure 6:**
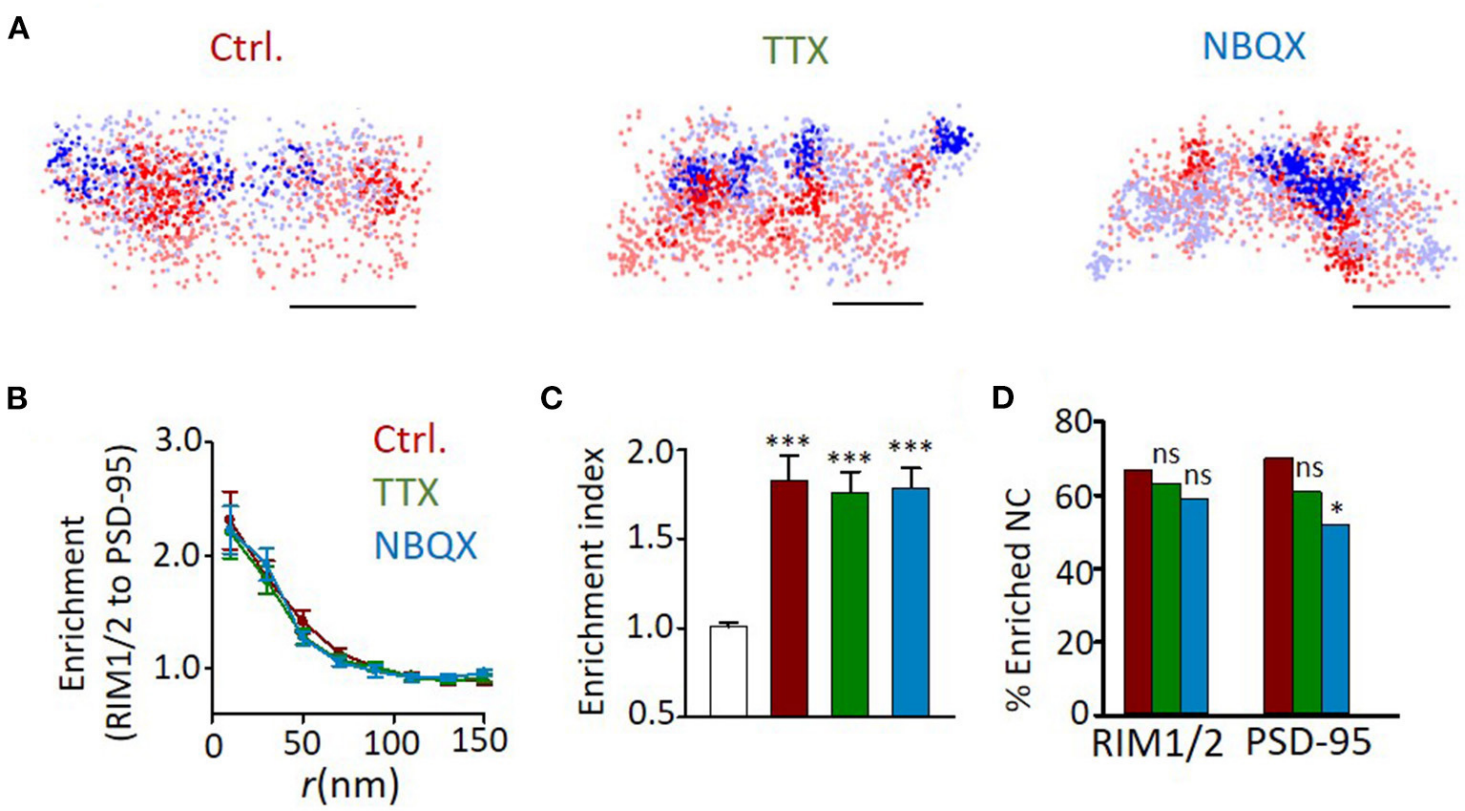
Effects of activity blockade on development of trans-synaptic nano-alignment. **(A)** Example distribution maps of both RIM1/2 (blue) and PSD-95 (red) with nanoclusters highlighted with thick color. Scale bars, 200 nm. **(B)** Normalized local density of RIM1/2 along with distances from PSD-95 nanoclusters for synapses from DIV18 cultures with different activities. **(C)** Averaged enrichment of RIM1/2 within 50 nm from peaks of PSD-95 nanoclusters (*n* = 60, 40, 51, and 49 nanoclusters). The open bar represents the enrichment indices of synapses with the position of nanoclusters randomized within synaptic clusters. **(D)** Fraction of nanoclusters that were enriched with protein across the cleft. *p < 0.05, ***p < 0.001, one-way ANOVA with Tukey's multiple comparisons in **(C)**, *z*-test in **(D)**. Also refer to Supplementary Table 1 for more statistical details. All experiments were repeated ≥3 times.

We further examined whether the formation of trans-synaptic nano-alignment depends on synaptic activity. We found that neither TTX nor NBQX treatment had a significant effect on the averaged enrichment between RIM1/2 and PSD-95 (Figures 6A–C). The lack of changes in general heterogeneity of protein organizations and the averaged nanoscale trans-synaptic enrichments is well-consistent with the electrophysiological result that there was no significant change in either the frequency or amplitude of mEPSCs (Supplementary Figure 4). However, the percentage of nanoclusters (NCs) that were enriched with their counter partners showed a reducing trend on activity blockade, with the difference significant for the proportion of enriched PSD-95 nanoclusters between NBQX and control groups (Figure 6D; *p* = 0.025, *z*-test). Therefore, in spite of the similar average overall enrichment between RIM1/2 and PSD-95, there might be some nanoscale reorganizations that concentrate the proteins within a subset of nanocolumns, especially for the RIM1/2-to-PSD-95 enrichment.

## Discussion

In this study, we used super-resolution microscopy to examine the evolvement of subsynaptic protein architectures during the genesis and maturation of synapses in hippocampal neuronal cultures. We revealed a dramatic reorganization in subsynaptic protein distribution of glutamatergic synapses accompanying a 4–5-fold increase in synapse size from DIV7 to DIV18. These changes are largely synchronous and proportional in presynaptic and postsynaptic compartments. More importantly, synaptic activities could shape the development of these nano-organizations and transsynaptic alignments in complicated ways. These findings have revealed a coordinated remodeling at the subsynaptic scale in presynaptic and postsynaptic compartments during development and suggest that both intrinsic and active mechanisms take part in controlling the formation of those nano-organizations to tune the synaptic functions.

It is well-documented that presynaptic and postsynaptic compartments of mature synapses correlate structurally in bouton and spine sizes, biochemically in protein amounts, and functionally in release probability and quantal amplitude (Harris and Stevens, 1989; Schikorski and Stevens, 1997; Nusser et al., 1998; Takumi et al., 1999; Thiagarajan et al., 2005; Regalado et al., 2006; Tokuoka and Goda, 2008; Kay et al., 2011; Holderith et al., 2012; Fisher-Lavie and Ziv, 2013; Rollenhagen et al., 2018; Holler et al., 2021). Our study has performed a systematic examination of the presynaptic and postsynaptic protein organizations at different developmental stages with super-resolution imaging methods. Our approach enables us to quantify not only the general synaptic properties, including the amount and cluster volume of key synaptic proteins but also the properties of subsynaptic nano-organizations. Consistent with an earlier pioneer study (Kay et al., 2011), we found a strong correlation between structural properties at the synapse scale, and this correlation is pronounced at every developmental stage, even after an activity blockade. These findings suggest that the coordinated development of general synaptic architecture does not require neuronal activity and is governed more likely by intrinsic mechanisms.

However, for the subsynaptic organizations which are thought to strongly modulate the action potential-dependent transmission, only the mature synapses show a significant correlation between the subsynaptic heterogeneity in presynaptic and postsynaptic compartments. Although details are still lacking, these nanocluster organizations are generally thought to result from multivalent interactions between proteins which lead to self-assembled condensates *via* phase separations in both presynaptic and postsynaptic compartments (Banani et al., 2017; Zeng et al., 2018, 2019; Wu et al., 2019), and the alignment between these condensates depends on protein interactions mediated by trans-synaptic adhesion molecules including neuroligins and LRRTM2 (Haas et al., 2018; Ramsey et al., 2021). Larger synapses generally have more presynaptic and postsynaptic proteins (Nusser et al., 1998; Holderith et al., 2012; Fisher-Lavie and Ziv, 2013); therefore, more multivalent interactions, stronger self-assembly, and higher internal heterogeneity in synaptic clusters could be expected. This is consistent with our finding that larger synapses show a higher degree of heterogeneity, regardless of the developmental stages. However, when we excluded the effect of the cluster size or protein amount by comparing only larger synapses with similar volumes, mature cultures showed significantly more subsynaptic heterogeneity. These data suggest that the evolvement, as well as the presynaptic and postsynaptic correlation of subsynaptic nano-organizations, requires active modulating mechanisms other than self-organization.

Accumulating studies suggest that spontaneous and action-potential-evoked transmitter release employs segregated vesicle pools and activates different groups of postsynaptic receptors (Reese and Kavalali, 2016; Crawford et al., 2017; Chanaday and Kavalali, 2018). Consistently, we found previously that the evoked release sites are more confined in the vicinity of RIM1/2 nanoclusters, while the spontaneous release sites distribute more broadly within the bouton (Tang et al., 2016), similar to the asynchronous release (Mendonça et al., 2021). Therefore, the simulations predict that the transsynaptic alignment directly modulates the strength of evoked transmission but not the amplitudes of spontaneous miniature currents (MacGillavry et al., 2013). This is well-consistent with the electrophysiological recordings in neurons with the alignment disrupted in response to LRRTM2 cleavage (Ramsey et al., 2021). Both miniature and evoked currents were reduced when the alignment was reduced by the expression of a truncated NL1 (Haas et al., 2018), but this may result from changes in presynaptic release probability by NL1 disturbance (Peixoto et al., 2012). Together, our finding that the development of transsynaptic alignment continued through DIV18 indicates an ongoing adjustment of action-potential (AP)-evoked transmission at this stage after the spontaneous transmission has saturated by DIV14 (Cottrell et al., 2000; Chanda et al., 2017). However, due to our limited understanding of the specific functional relevance of these subsynaptic organizations, so far it is hard to make valid predictions on the impacts on the synaptic transmission based on our structural quantifications.

Synaptic activity plays an important role in regulating synaptic morphology, transmission strength, neuronal membrane properties, and neural circuit refinement (Maletic-Savatic et al., 1999; Nick and Ribera, 2000; Groc et al., 2002; Walmsley et al., 2006; West and Greenberg, 2011; Chaudhury et al., 2016). We examined the effect of synaptic activity on subsynaptic protein nano-organizations and found a set of complicated impacts of activity blockade on the synaptic nano-architectures. Despite that reduced activity resulted in larger synaptic volumes and increased nanocluster numbers, which is largely consistent with previous studies with similar treatments (MacGillavry et al., 2013; Glebov et al., 2017), neither the general heterogeneity of protein organizations nor the averaged nanoscale trans-synaptic enrichments were significantly altered by activity blockade. These results are consistent with the electrophysiological result that there was no significant change in either the frequency or amplitude of mEPSCs. These data argue against the hypothesis that activity-dependent mechanism is directly involved in establishing nanocolumns. Instead, these results favor a self-organization model for nanocolumn organizations, and the neuronal activity may exert its influence through changing the amount of protein components. However, besides RIM1/2 and PSD-95, in this study, we examined, there are many other proteins involved in the construction of nanocolumns and the modulation of synaptic transmission in both presynaptic and postsynaptic compartments. It is very possible that other MAGUK members and scaffolding molecules are modulated by activities. In fact, the reduction in the fraction of enriched PSD-95 nanoclusters on AMPAR blockade suggests a mechanism that actively strengthens some nanocolumns while dismantling others. A similar process was observed previously after the induction of NMDA receptor-dependent long-term depression (Tang et al., 2016). This may result from changes in synaptic activity-dependent gene transcriptions (Yap and Greenberg, 2018) and activity-dependent specific rearrangements in a targeted synaptic protein interaction network (Lautz et al., 2018). The detailed mechanism needs further investigation.

Activity deprivation has been found to significantly increase the puncta size and the amount of synaptic proteins (Noritake et al., 2009; Sun and Turrigiano, 2011; Letellier et al., 2014; Venkatesan et al., 2020) through a homeostatic plasticity mechanism (Turrigiano et al., 1998; Turrigiano and Nelson, 2004). However, the changes in RIM1/2 and PSD-95 in our results are not as strong. This may result from the long duration of the activity blockade we used (8 days). The major aim of our activity blockade experiments is to test its impact on the trans-synaptic alignment. Since the largest change in enrichment index is between synapses on DIV10 and on DIV18 (synapses on DIV7 are not fully formed), we selected this period to test the effects of activity blockade. This is quite different from the 48 h treatment protocol that most people use in studies on homeostatic plasticity. More complex processes may have taken place during this prolonged treatment. There is less overlap in newly synthesized proteins for cultured hippocampal neurons within 2 and 24 h after TTX treatment (Schanzenbächer et al., 2018), and the neurons at different developmental stages undergo distinct synaptic functional reorganizations (Han and Stevens, 2009), suggesting a strong temporal dependency of the impact of activity deprivation on synapses. As another example, for homeostatic modulation of synaptic transmission by transcranial direct current stimulation in the motor cortex of healthy humans, two sets of 5-min stimulations could induce opposite plasticity depending on the time intervals (Fricke et al., 2010). Consistent with this, after the long activity blockade, neither frequency nor amplitudes of mEPSCs showed significant changes, which is quite different from the dramatic increase in these parameters in response to a 1 or 2 days incubation (Han and Stevens, 2009). More future studies are required to figure out the duration-dependent modulation of synapses in response to activity blockade.

During synaptogenesis in cultured hippocampal neurons, the presynaptic proteins accumulate before postsynaptic receptors and PSD-95 (Li and Sheng, 2003). This indicates that presynaptic differentiation precedes postsynaptic development. However, in our results, the evolvement of either the general cluster properties or features of subsynaptic nano-organizations seems synchronous between presynaptic and postsynaptic compartments. Two technical limitations may attenuate our ability of detecting the potential precedence in development. First, the temporal resolution in our design is not optimized for this purpose. This is exacerbated by the fact that the development of dissociated neurons varies with culture density and conditions. Second, we pick up synapses based on colocalization of presynaptic and postsynaptic clusters, which would exclude those early structures with one side preceding the other. However, when comparing the dependency of protein heterogeneity on cluster size, we found that both RIM1/2 and GluA1 show a significant difference between immature and mature synapses while the heterogeneity of PSD-95 is decided solely by cluster volume and independent of maturation. This result indicated a unique role of PSD-95 in organizing the subsynaptic architectures. It will be important for future studies to validate this constructing sequence of nanocolumns *in vivo* and examine the detailed molecular mechanisms.

## Data Availability Statement

The raw data supporting the conclusions of this article will be made available by the authors, without undue reservation.

## Ethics Statement

The animal study was reviewed and approved by Institutional Animal Care and Use Committees at the University of Science and Technology of China (USTC) and the Chinese Academy of Sciences (CAS).

## Author Contributions

S-YS, X-WL, and RC conducted the experiments. S-YS, YZ, and A-HT performed the analysis and prepared the figure. NS and A-HT designed the project and wrote the manuscript. All authors contributed to the article and approved the submitted version.

## Funding

The authors acknowledge National Key Research and Development Program of China 2021ZD0202503, National Natural Science Foundation of China (31872759, 31871032), the USTC Cultivation Fund for Innovation Team, Open Project from the State Key Laboratory of Genetic Resources and Evolution GREKF19-09 to A-HT, Strategic Priority Research Program of the Chinese Academy of Sciences XDPB17, Yunnan Applied Basic Research Projects 2019FA008 and 2019FJ003, and CAS Light of West China Program xbzg-zdsys-201913 to NS.

## Conflict of Interest

The authors declare that the research was conducted in the absence of any commercial or financial relationships that could be construed as a potential conflict of interest.

## Publisher's Note

All claims expressed in this article are solely those of the authors and do not necessarily represent those of their affiliated organizations, or those of the publisher, the editors and the reviewers. Any product that may be evaluated in this article, or claim that may be made by its manufacturer, is not guaranteed or endorsed by the publisher.
