# Supplementary material for: Correlative Assembly of Subsynaptic Nanoscale Organizations During Development
Source: Front Synaptic Neurosci. 2022 May 24;14:748184. doi: 10.3389/fnsyn.2022.748184 (PMC9171000; doi:10.3389/fnsyn.2022.748184)
Supplement: Supplementary file 1 [file Data_Sheet_1.PDF]

Supplementary Table 1. Statistical results.

1. Developmental changes

Synaptic cluster volume (Figure 1E, F, G):

One Way ANOVA with Tukey's multiple comparisons test

**GluA1**	N	P	Summary
DIV6-8 vs DIV9-11	63, 32	0.5864	ns
DIV6-8 vs DIV14-15	63, 32	<0.0001	****
DIV6-8 vs DIV18-20	63, 29	<0.0001	****
DIV9-11 vs DIV14-15	32, 32	<0.0001	****
DIV9-11 vs DIV18-20	32, 29	<0.0001	****
DIV14-15 vs DIV18-20	32, 29	0.4023	ns
**RIM1/2**			
DIV6-8 vs DIV9-11	98, 79	0.0418	*
DIV6-8 vs DIV14-15	98, 85	<0.0001	****
DIV6-8 vs DIV18-20	98, 69	<0.0001	****
DIV9-11 vs DIV14-15	79, 85	<0.0001	****
DIV9-11 vs DIV18-20	79, 69	<0.0001	****
DIV14-15 vs DIV18-20	85, 69	0.0116	*
**PSD-95**			
DIV6-8 vs DIV9-11	25, 48	0.5661	ns
DIV6-8 vs DIV14-15	25, 59	0.0005	***
DIV6-8 vs DIV18-20	25, 27	<0.0001	****
DIV9-11 vs DIV14-15	48, 59	0.006	**
DIV9-11 vs DIV18-20	48, 27	<0.0001	****
DIV14-15 vs DIV18-20	59, 27	0.0883	ns

Normalized density within nanocluster (Figure 2H, I, J):

One Way ANOVA with Tukey's multiple comparisons test

**GluA1**	N	P	Summary
DIV6-8 vs DIV9-11	50, 22	0.0668	ns
DIV6-8 vs DIV14-15	50, 30	0.0001	***
DIV6-8 vs DIV18-20	50, 27	<0.0001	****
DIV9-11 vs DIV14-15	22, 30	0.5316	ns
DIV9-11 vs DIV18-20	22, 27	0.0592	ns
DIV14-15 vs DIV18-20	30, 27	0.5546	ns
**RIM1/2**			
DIV6-8 vs DIV9-11	75, 77	<0.0001	****
DIV6-8 vs DIV14-15	75, 85	<0.0001	****
DIV6-8 vs DIV18-20	75, 69	<0.0001	****
DIV9-11 vs DIV14-15	77, 85	0.1232	ns
DIV9-11 vs DIV18-20	77, 69	<0.0001	****
DIV14-15 vs DIV18-20	85, 69	0.0004	***
**PSD-95**			

DIV6-8 vs DIV9-11	22, 46	<0.0001	****
DIV6-8 vs DIV14-15	22, 57	<0.0001	****
DIV6-8 vs DIV18-20	22, 27	<0.0001	****
DIV9-11 vs DIV14-15	46, 57	>0.9999	ns
DIV9-11 vs DIV18-20	46, 27	0.0053	**
DIV14-15 vs DIV18-20	57, 27	0.0034	**

NC volume (Figure 2H, I, J):
One Way ANOVA with Tukey's multiple comparisons test

**GluA1**	N	P	Summary
DIV6-8 vs DIV9-11	42, 26	0.9756	ns
DIV6-8 vs DIV14-15	42, 41	<0.0001	****
DIV6-8 vs DIV18-20	42, 59	<0.0001	****
DIV9-11 vs DIV14-15	26, 41	0.0004	***
DIV9-11 vs DIV18-20	26, 59	<0.0001	****
DIV14-15 vs DIV18-20	41, 59	0.9083	ns
**RIM1/2**			
DIV6-8 vs DIV9-11	77, 115	0.3095	ns
DIV6-8 vs DIV14-15	77, 181	<0.0001	****
DIV6-8 vs DIV18-20	77, 137	<0.0001	****
DIV9-11 vs DIV14-15	115, 181	0.0028	**
DIV9-11 vs DIV18-20	115, 137	<0.0001	****
DIV14-15 vs DIV18-20	181, 137	0.0329	*
**PSD-95**			
DIV6-8 vs DIV9-11	23, 64	0.8939	ns
DIV6-8 vs DIV14-15	23, 109	0.501	ns
DIV6-8 vs DIV18-20	23, 54	0.0326	*
DIV9-11 vs DIV14-15	64, 109	0.7799	ns
DIV9-11 vs DIV18-20	64, 54	0.0305	*
DIV14-15 vs DIV18-20	109, 54	0.1316	ns

NC number (Figure 2H, I, J):
One Way ANOVA with Tukey's multiple comparisons test

**GluA1**	N	P	Summary
DIV6-8 vs DIV9-11	71, 32	0.0138	*
DIV6-8 vs DIV14-15	71, 32	<0.0001	****
DIV6-8 vs DIV18-20	71, 29	<0.0001	****
DIV9-11 vs DIV14-15	32, 32	0.7561	ns
DIV9-11 vs DIV18-20	32, 29	0.0314	*
DIV14-15 vs DIV18-20	32, 29	0.2052	ns
**RIM1/2**			
DIV6-8 vs DIV9-11	98, 79	<0.0001	****
DIV6-8 vs DIV14-15	98, 85	<0.0001	****
DIV6-8 vs DIV18-20	98, 69	<0.0001	****

DIV9-11 vs DIV14-15	79, 85	<0.0001	****
DIV9-11 vs DIV18-20	79, 69	0.0001	***
DIV14-15 vs DIV18-20	85, 69	0.9955	ns
**PSD-95**			
DIV6-8 vs DIV9-11	25, 48	0.1469	ns
DIV6-8 vs DIV14-15	25, 59	<0.0001	****
DIV6-8 vs DIV18-20	25, 27	<0.0001	****
DIV9-11 vs DIV14-15	48, 59	0.0061	**
DIV9-11 vs DIV18-20	48, 27	0.0097	**
DIV14-15 vs DIV18-20	59, 27	0.9571	ns

$\overline{g_a}$ (r < 50) (Figure 3F):
One Way ANOVA with Tukey's multiple comparisons test

DIV7-10 vs DIV18	N	P	Summary
**GluA1**	20,19	<0.0001	****
**RIM1/2**	16,18	<0.0001	****
**PSD-95**	25,20	>0.9999	ns

Enrichment analysis (Figure 4F, I):
One Way ANOVA with Tukey's multiple comparisons test

**GluA1 to RIM1/2**	N	P	Summary
Rand. vs DIV6-8	85, 60	0.4515	ns
Rand. vs DIV9-11	85, 30	0.2518	ns
Rand. vs DIV14-15	85, 54	0.0301	*
Rand. vs DIV18-20	85, 54	<0.0001	****
DIV6-8 vs DIV9-11	60, 30	0.9626	ns
DIV6-8 vs DIV14-15	60, 54	0.7451	ns
DIV6-8 vs DIV18-20	60, 54	0.0126	*
DIV9-11 vs DIV14-15	30, 54	0.9971	ns
DIV9-11 vs DIV18-20	30, 54	0.2739	ns
DIV14-15 vs DIV18-20	54, 54	0.2922	ns
**RIM1/2 to PSD-95**			
Rand. vs DIV6-8	60, 25	0.9988	ns
Rand. vs DIV9-11	60, 77	0.343	ns
Rand. vs DIV14-15	60, 106	0.0079	**
Rand. vs DIV18-20	60, 49	0.0001	***
DIV6-8 vs DIV9-11	25, 77	0.8024	ns
DIV6-8 vs DIV14-15	25, 106	0.2038	ns
DIV6-8 vs DIV18-20	25, 49	0.0119	*
DIV9-11 vs DIV14-15	77, 106	0.5749	ns
DIV9-11 vs DIV18-20	77, 49	0.0283	*
DIV14-15 vs DIV18-20	106, 49	0.3569	ns

2. Effect of activity blockade

Synaptic cluster volume (Figure5B, F):

One Way ANOVA with Tukey's multiple comparisons test

**RIM1/2**	N	P	Summary
Ctrl vs TTX	40, 51	0.3557	ns
Ctrl vs NBQX	40, 69	0.0092	**
TTX vs NBQX	51, 69	0.233	ns
**PSD-95**			
Ctrl vs TTX	40, 51	0.1787	ns
Ctrl vs NBQX	40, 69	<0.0001	****
TTX vs NBQX	51, 69	0.0112	*

NC number (Figure5C, G):

One Way ANOVA with Tukey's multiple comparisons test

**RIM1/2**	N	P	Summary
Ctrl vs TTX	40, 51	0.8497	ns
Ctrl vs NBQX	40, 69	0.0507	ns
TTX vs NBQX	51, 69	0.1369	ns
**PSD-95**			
Ctrl vs TTX	40, 51	0.2519	ns
Ctrl vs NBQX	40, 69	0.0039	**
TTX vs NBQX	51, 69	0.2137	ns

Normalized density:

One Way ANOVA with Tukey's multiple comparisons test

**RIM1/2**	N	P	Summary
Ctrl vs TTX	40, 51	0.1683	ns
Ctrl vs NBQX	40, 69	0.3327	ns
TTX vs NBQX	51, 69	0.8488	ns
**PSD-95**			
Ctrl vs TTX	39, 50	0.5203	ns
Ctrl vs NBQX	39, 68	0.9938	ns
TTX vs NBQX	50, 68	0.4922	ns

Enrichment analysis (Figure 6C):

One Way ANOVA with Tukey's multiple comparisons test

**RIM1/2 to PSD-95**	N	P	Summary
Rand. vs Ctrl	60, 51	0.0001	***
Rand. vs TTX	60, 78	<0.0001	****
Rand. vs NBQX	60, 120	<0.0001	****
Ctrl vs TTX	51, 78	0.9823	ns
Ctrl vs NBQX	51, 120	0.9943	ns
TTX vs NBQX	78, 120	0.9982	ns
