# Supplementary material for: Correlative Assembly of Subsynaptic Nanoscale Organizations During Development
Source: Front Synaptic Neurosci. 2022 May 24;14:748184. doi: 10.3389/fnsyn.2022.748184 (PMC9171000; doi:10.3389/fnsyn.2022.748184)
Supplement: Supplementary file 2 [file Data_Sheet_2.PDF]

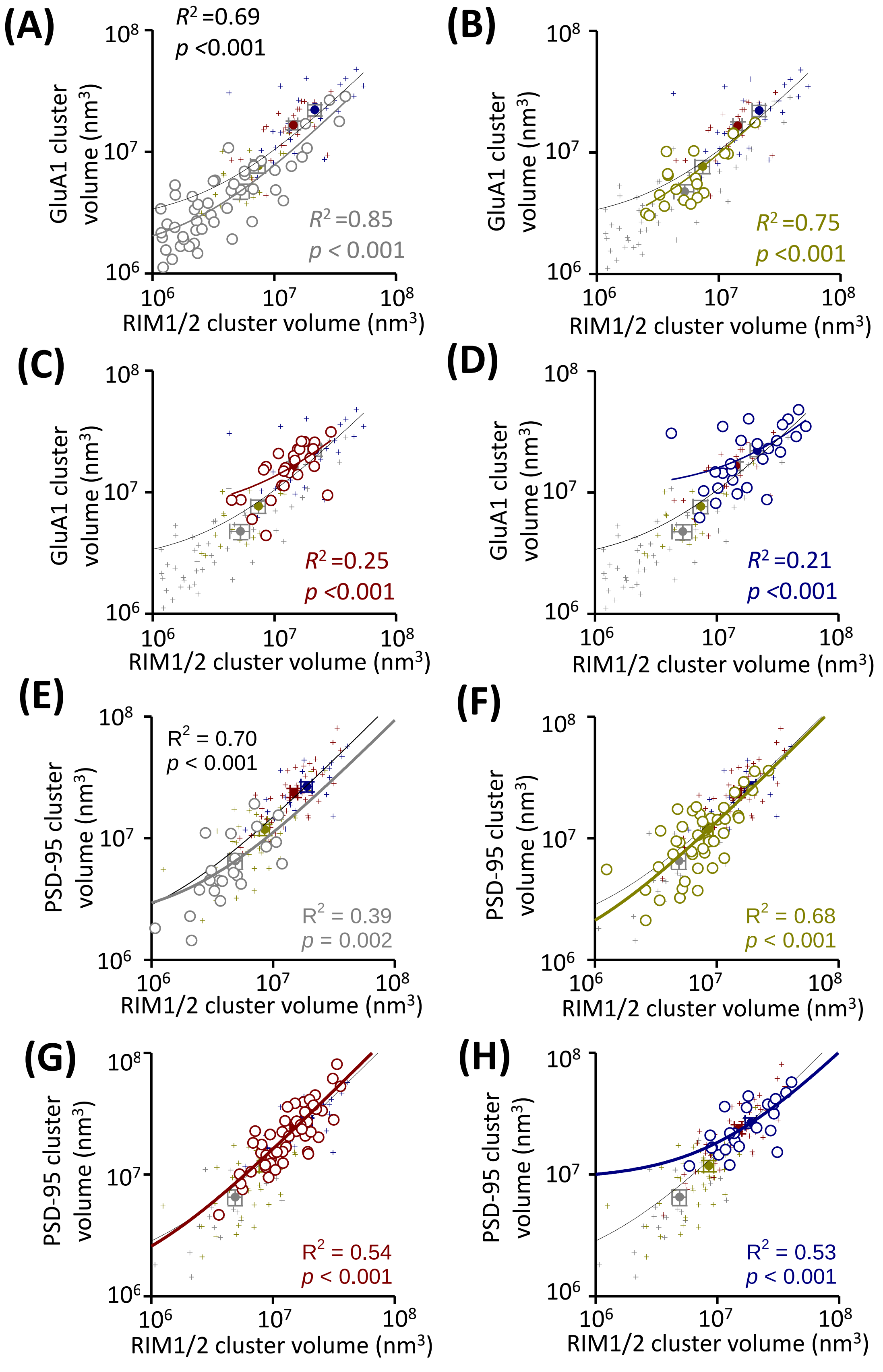

Supplementary Figure 1. Correlation between pre- and postsynaptic cluster volume. ***A-D***, Correlations between the synaptic cluster volume of GluA1 and RIM1/2 clusters within the same synapses. Thin line was linear regression of data from all groups. Thick lines were regressions of data from DIV7 (***A***), DIV10 (***B***), DIV14 (***C***), and DIV18 (***D***). ***E-H***, Correlations between the synaptic cluster volume of PSD-95 and RIM1/2 within the same synapses. See also Supplementary Table 1 for more details on correlations and statistics. All experiments were repeated on ≥3 sets of cultures.

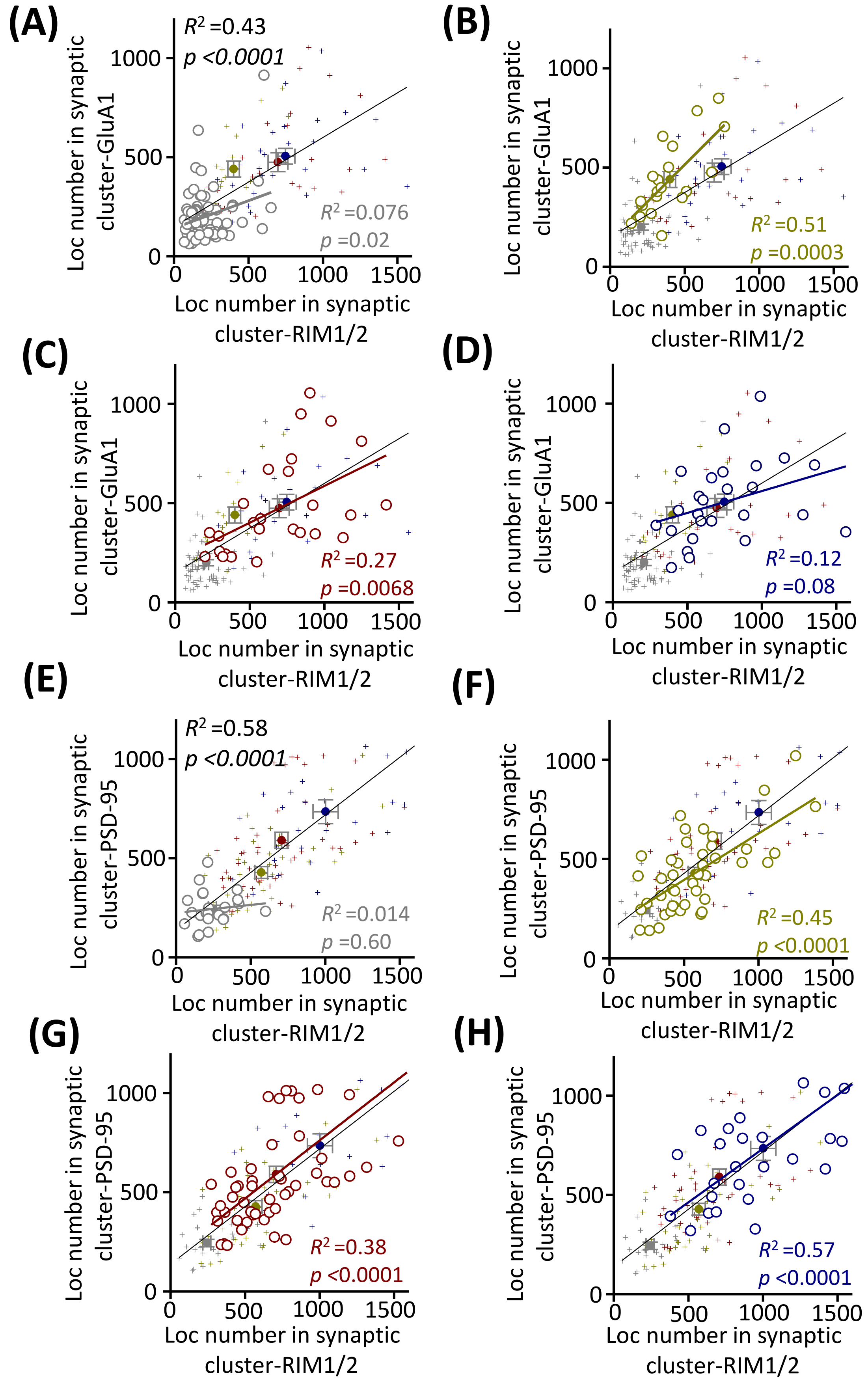

Supplementary Figure 2. Correlation between localization numbers in pre- and postsynaptic clusters. ***A-D***, Correlations between localization numbers of GluA1 and RIM1/2 within the same synapses. Thin line was linear regression of data from all groups. Thick lines were regressions of data from DIV7 (***A***), DIV10 (***B***), DIV14 (***C***), and DIV18 (***D***). ***E-H***, Correlations between the localization numbers of PSD-95 and RIM1/2 within the same synapses. See also Supplementary Table 1 for more details on correlations and statistics. All experiments were repeated on ≥3 sets of cultures.

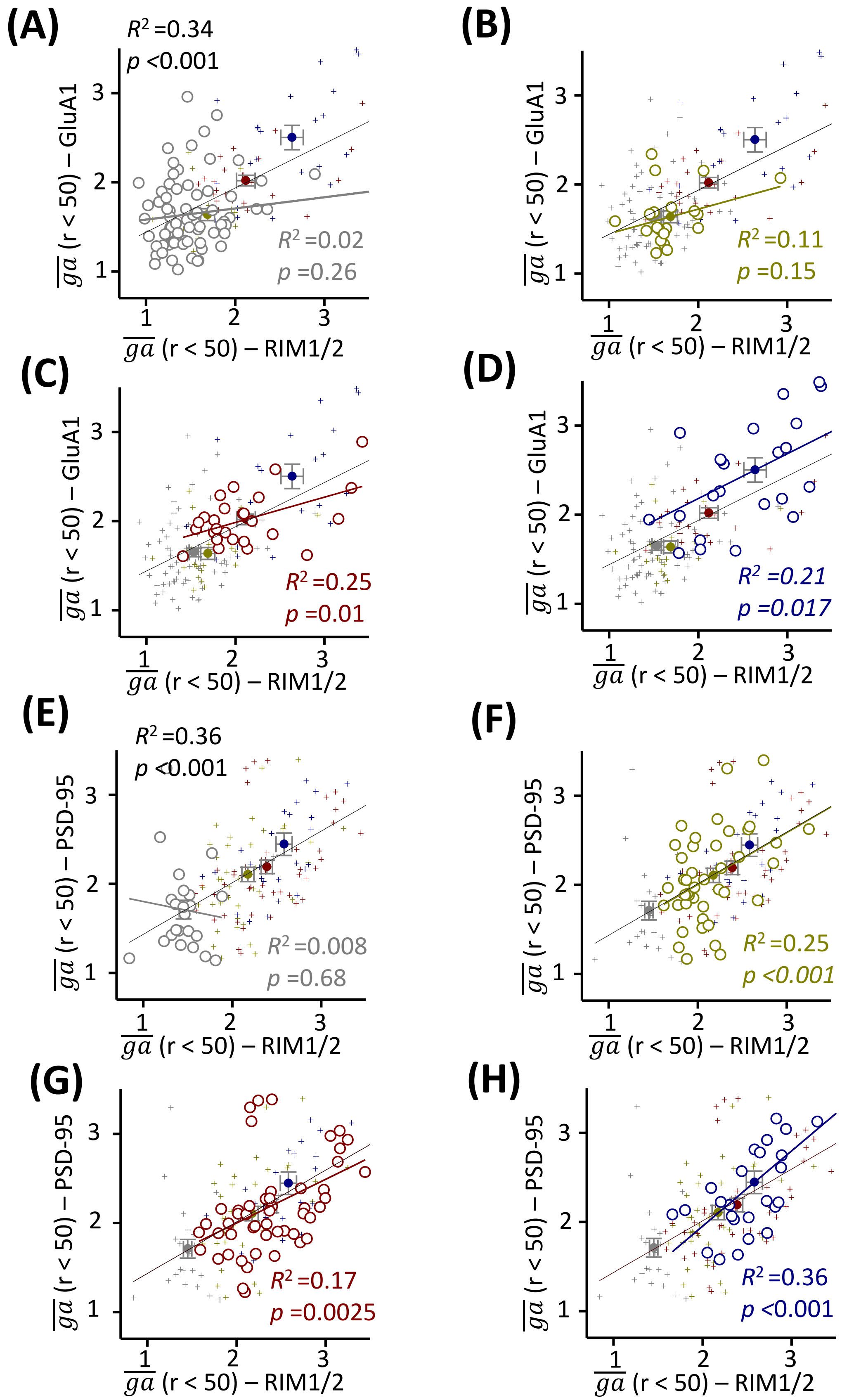

Supplementary Figure 3. Correlation between pre- and postsynaptic protein heterogeneity in the same synapses. ***A-D***, Correlations between the $\overline{ga}$ (r < 50) of GluA1 and RIM1/2 clusters within the same synapses. Thin line was linear regression of data from all groups. Thick lines were linear regressions of data from DIV7 (***A***), DIV10 (***B***), DIV14 (***C***), and DIV18 (***D***). ***E-H***, Correlations between the $\overline{ga}$ (r < 50) of PSD-95 and RIM1/2 clusters within the same synapses. See also Supplementary Table 1 for more details on correlations and statistics. All experiments were repeated on ≥3 sets of cultures.

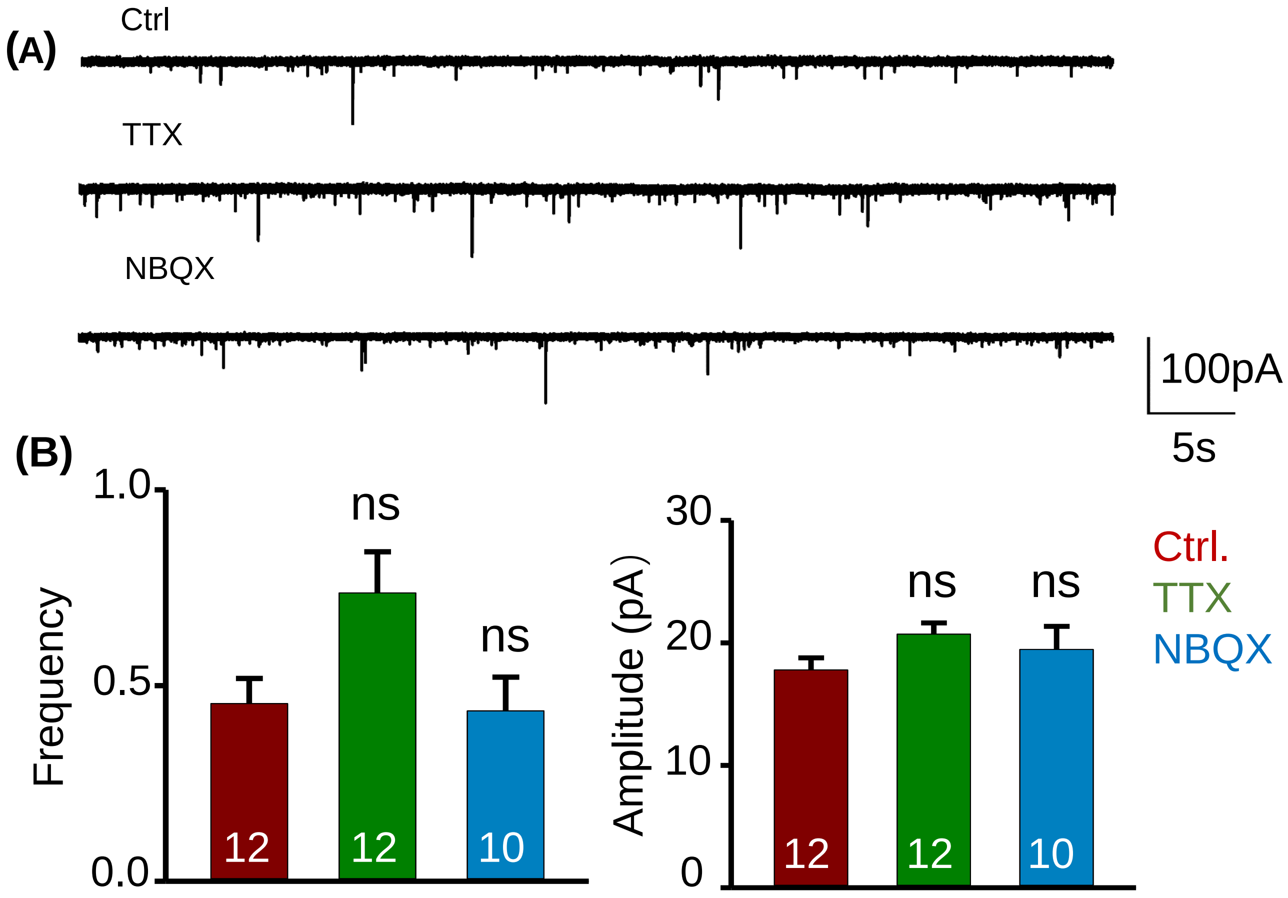

Supplementary Figure 4. (A) Example traces from Ctrl, TTX, and NBQX group respectively. Scale bar: 100pA, 5s. (B) mEPSC frequency did not change or had a slightly increase after drug treatment. (Ctrl: 0.45 ± 0.06, n = 12; TTX: 0.74 ± 0.10, n = 12, P = 0.0623; NBQX: 0.44 ± 0.08, n = 10, P = 0.9883; one-way ANOVA with Tukey's multiple comparisons, vs Ctrl); mEPSC amplitude did not change or had a slightly increase after drug treatment. (Ctrl: 17.78 ± 0.10, n = 12; TTX: 20.73 ± 0.91, n = 12, P = 0.2213; NBQX: 19.47 ± 1.88, n = 10, P = 0.6260; one-way ANOVA with Tukey's multiple comparisons, vs Ctrl). Error bars were SEM.
